# Outcome of Induction of Labour in Nulliparous Women Following Replacement of Cervidil with Prostin

**DOI:** 10.1100/2012/325968

**Published:** 2012-04-30

**Authors:** Abhijit Basu, Stephen Elgey, Mano Haran

**Affiliations:** ^1^Department of Obstetrics and Gynaecology, Logan Hospital, Cnr Armstrong and Loganlea Road, Meadowbrook, QLD 4131, Australia; ^2^Department of Obstetrics and Gynaecology, School of Medicine Griffith University, Southport, Gold Coast, QLD 4222, Australia

## Abstract

This study at the Logan Hospital, Australia, compared the outcome of induction of labour (IOL) in nulliparous women following replacement of Cervidil with Prostin. Eighty-two nulliparous women were identified for this retrospective cohort study over a period of three months on either side of the changed practice. Forty-four women received Prostin and 38 received Cervidil. Baseline characteristics were similar amongst the groups including maternal age, mean gestational age, and modified Bishop's score at the commencement of IOL. The incidence of amniotomy, oxytocin augmentation of labour, and rate of epidural use did not differ significantly between the groups. The mean time to delivery (vaginally or abdominally) showed a significant difference, with women receiving Prostin delivering earlier than those having Cervidil (*P* = 0.018). Women receiving Prostin were more likely to have assisted vaginal delivery compared to the Cervidil group (*P* = 0.04).

## 1. Introduction

Induction of labour (IOL) is one of the commonest intervention in obstetrics. It is estimated that in the developed world, at least 19.8% of all labours are induced [1]. Vaginal prostaglandin E2 has been shown to be efficacious to prepare the cervix [2] for IOL. Various preparations of prostaglandin are available differing in their effectiveness, side effects [3], and price [4]. The preparation most commonly used for IOL is the shorter acting Dinoprostone vaginal gel (Prostin). Recently longer-acting Dinoprostone preparations (Cervidil and Propess) with retrieval system have become available which have been successfully used for IOL. Nevertheless, some studies [4] show that longer-acting preparations do not reduce time to delivery or improve any birth outcome compared to the shorter acting gel which is also considered more cost effective [5]. It has been noted [3] that more than one dose of the long acting preparation is needed to achieve amniotomy compared to the shorter acting one. The only consistent benefit seems to be the lesser number of vaginal examinations with the long acting preparations.

A potential risk of IOL is failure of this intervention resulting in delivery by Caesarean section (CS) [6]. Recent findings [1] show that following IOL the emergency CS rate may be as high as 22%. Others [7] have noted that IOL in nulliparous women at term, with or without medical or obstetric complications, significantly increases the chance of CS. CS rates in primiparous women are a variable life-adjusted (VLAD) clinical indicator in Queensland, Australia. VLAD is a statistical control chart used by the Queensland Health to better monitor treatment outcomes across the state [8]. The VLAD tool records patient outcomes for any clinical indicator at a particular facility identifying variation against the state averages. It is believed that any variation may reflect shift in practice or treatment quality.

Logan Hospital in Queensland has been using Cervidil for IOL in nulliparous women for three years till June 2010. There was a change in practice for IOL from July 2010 when Cervidil was replaced with Prostin. The purpose of this retrospective cohort study was to compare the outcome of IOL three months after the change in practice with regards to the time interval between the initiation of the induction process and the birth of the baby and impact on the mode of delivery.

## 2. Methods

Logan Hospital is a secondary level general hospital in Queensland, Australia within the Metro South Health Service District with an annual delivery rate of 3500. It serves a population of around 300,000 and is one of the most multicultural districts in Australia representing some 185 ethnicities. The population is young with a median age of 33 years, and 30% of the population is aged between 25 and 44 years [9].

The women for this retrospective cohort study were identified from the IOL database which is an in-hospital electronic record system. The data was obtained from the hospital electronic patient record system (ERIC) which archives every patient record entry and obviates the need for any hard copy. Information was collected on the outcome of IOL in nulliparous women induced in the last three months of Cervidil use (May–July, 2010) and the first three months after the change to Prostin (August–October, 2010). This time frame was specifically chosen to minimize the influence of operator skill as doctors who work on the birth suite, do so for a period of twelve weeks at a stretch, working alternate weeks to comply with the directive on hours worked. The departmental protocol till 31st of July 2010 for IOL in a nulliparous woman with a singleton foetus in cephalic presentation regardless of the gestation is described below.

Planned IOL was to commence at 1400 hrs. An initial vaginal examination was done to determine the modified Bishop's score [10]. If the score was greater than 6 then the woman was to have amniotomy next morning at 0600 followed by augmentation with oxytocin infusion if necessary. If the modified Bishop's score was less than 7, then labour was induced by placing Cervidil (retrievable controlled release 10 mg Dinoprostone pessary releasing 0.3 mg Dinoprostone/hour) into the posterior vaginal fornix. Electronic foetal monitoring was done for an hour after insertion of Cervidil. The next assessment would be at 0600 the following morning or earlier if there was any suggestion of active labour. If cervix was unfavourable for amniotomy then consultant advice was sought regarding further prostaglandin gel.

This practice was changed from 1st of August 2010 when Cervidil was replaced with Prostin. A 2 mg dose was placed into the posterior fornix at 1400 hours followed by electronic foetal monitoring for 1 hour. A reassessment was done six hours later, and a further 1 mg intravaginal gel was inserted if the modified Bishop's score was less than 7. Amniotomy was done if this score was greater than 6. If the second dose of intravaginal gel was administered then, the next examination was at 0600 the following morning to consider amniotomy or the need for a further third 1 mg dose of intravaginal gel. Regardless of the change in practice, failed IOL was defined as inability of the agent to dilate the cervix to enable amniotomy, or failure of the cervix to dilate beyond 4 cm despite at least 10 hours of a titrated oxytocin infusion.

Though this study was based on an audit of a change in departmental practice, an ethical approval was obtained from the local Human Research Ethics Committee of the Metro South Health Service District of Queensland Health of Queensland Government, Australia.

Data was entered on a Microsoft Excel spreadsheet. Statistical analysis was done using Analyse-it (version 2.20) statistics software for Microsoft Excel. The groups were compared using contingency table and chi-square analysis for categorical and binary values. Continuous variables were analysed by a *t*-test. Two-sided *P* values are reported for all tests. Values 0.05 or less were regarded as significant.

## 3. Results

There were 83 women who were eligible for inclusion, 44 in the Prostin group, and 39 in the Cervidil group. One case was excluded from the Cervidil group (*n* = 38) as the IOL was commenced with Cervidil, postponed, and then recommenced 3 days later with Prostin making classification impossible.

The baseline characteristics were similar amongst both groups as shown in Table 1 including maternal age, mean gestational age at IOL, and the modified Bishop's score at the commencement of IOL. As expected, the commonest indication for IOL in both groups was prolonged pregnancy beyond 40 weeks of gestation. Other common indications for IOL were hypertensive diseases in pregnancy and gestational diabetes mellitus.

Outcome of IOL in the two groups is detailed in Table 2. The number of women requiring amniotomy and oxytocin infusion to cause effective contractions and delivery did not differ between the two groups. The incidence of epidural analgesia use during labour did not vary between the two groups either. Four women were recorded as having a failed IOL, one in the Prostin group and three in the Cervidil group (RR 0.29, 95%, CI 0.03–2.65, *P* = 0.51), but this difference was not significant. There was no difference in the incidence of caesarean section (RR 0.65, 95% CI 0.35–1.19, *P* = 0.24) between the Prostin and Cervidil groups. There was also no statistically significant difference in the number of women needing an operative delivery (either a caesarean section or assisted vaginal delivery) between the two groups (RR 1.12, 95% CI 0.76–1.66, *P* = 0.72). However, there was a significant difference in the number of women requiring instrumental delivery, with those receiving Prostin more likely to need assisted vaginal delivery (RR 3.0, 95% CI 1.1–8.4, *P* = 0.04). The mean time to delivery for all women also showed a significant difference, with women receiving Prostin delivering earlier on average than those having Cervidil (21.1 versus 29.6 hrs *P* = 0.018) irrespective of the mode of delivery.

## 4. Discussion

IOL is a common obstetric intervention. It is therefore considered an important clinical indicator both by the Australian council of Healthcare standards [11] and Core maternity indicator project Australia [12]. Moreover, failed induction of labour results in caesarean section which again is a VLAD indicator especially in nulliparous women. Hence, judicious use and selection of agents apart from using strict criteria for IOL should underpin this intervention. It is argued that IOL can place more strain on birthing suite workload than spontaneous labour [1]. Therefore, timing of IOL is also of importance which again is related to the induction delivery interval.

It appears from this small retrospective study that Prostin is more likely to achieve a quicker delivery, regardless of the mode of delivery, in nulliparous women following IOL than Cervidil. This was not observed by others [5] who however, used a different proprietary long-acting retrievable preparation. An earlier larger but similar retrospective cohort study [4] had noted that nulliparous women induced with the longer acting preparation had significantly longer median insertion to vaginal birth interval. The finding from this study is likely to have a favourable impact especially for elective IOL which is known to affect the workload in birth suite. The concern amongst women undergoing IOL about the length of labour is well known [13]. Hence, it is believed [14] that women are likely to value a reduction in the interval between induction and delivery. Therefore the finding that the preparation which is associated with a significant reduction in the delivery time irrespective of the mode of delivery being used in their care is likely to go down favourably with the women undergoing IOL.

Rate of caesarean section in primiparous women, a VLAD indicator, does not appear to be increased with either Prostin or Cervidil, and hence there was no impact on the overall performance. The indications for caesarean sections in both groups included acute foetal bradycardia not responding to intrauterine resuscitative measures, foetal acidaemia determined by foetal scalp lactate and/or pH and base excess, arrest of labour in the first stage despite augmentation with oxytocins, and failed assisted vaginal delivery apart from failed induction. However, the numbers in each of these groups were too small to calculate any statistical significance. There was no incidence of hyperstimulation with either preparation.

This study, however, shows that using Prostin for IOL is more likely to be associated with assisted vaginal delivery than Cervidil though the overall incidence of operative deliveries (assisted vaginal delivery and caesarean section) is no different with the use of either agent. This greater incidence of assisted vaginal delivery with Prostin compared to Cervidil was not addressed in a previous study [4]. It is uncertain whether this greater likelihood of successful assisted vaginal delivery was due to the fact that the department had significantly more doctors in their advanced years of training in the second half of the year than in the first. There is good evidence of higher incidence of caesarean section at full dilatation with lesser experienced operators [15]. During the study period, there was only one instance of failed assisted vaginal delivery which resulted in a caesarean section at full dilatation. Incidentally, most of the caesarean sections done in the 3 months of Cervidil use were done in the first stage of labour and hence less likely to have been influenced by operator skill.

This study nevertheless has several limitations. The numbers are small, and the data relates to a single birthing unit in one hospital. The time frame used in this study is only six months with three months on either side of change in practice. The study is not a randomised trial but retrospective data analysis. It is also difficult to explain why women having IOL with Prostin achieved an earlier delivery than those who were induced with Cervidil. The basic mechanism of action with either preparation is essentially the same being mediated through a combination of reduced collagen concentration and dissociation of collagen fibrils by activation of the collagenase enzyme apart from the alterations in glycosaminoglycan composition and hydration [16]. One reason may be the additive effects of repeated doses of Prostin compared to the slow-release Cervidil preparation that allowed an earlier amniotomy and oxytocin augmentation. This would have allowed a diagnosis of failed IOL or an operative delivery earlier. However, these interventions did not reach any statistical significance between the two arms.

## 5. Conclusion

In conclusion, this is a small study comparing the outcome of IOL in nulliparous women following a change in clinical practice after replacement of a long-acting vaginal prostaglandin E2 by a shorter-acting preparation. IOL with the shorter acting preparation was likely to result in a quicker delivery regardless of the mode of delivery (operative or vaginal), a feature likely to be appreciated by the women undergoing the intervention. This albeit was associated with a higher assisted vaginal delivery rate, but there was no significant difference in the caesarean section or overall (spontaneous and assisted) vaginal delivery rates.

##  Conflict of Interests 

The authors declare that they have no conflicts of interests.

## Figures and Tables

**Table 1:** Baseline characteristics of the women in the Prostin and Cervidil induction agent groups.

Characteristic	Prostin (*N* = 44)	Cervidil (*N* = 38^§^)	*P*
Age (yrs)	24.5	25.2	0.465
Gestation (weeks)	40.2	40.2	0.978
BMI			
Bishops score (median)	2	2	1.000

Primary Indication	Number (%)		

Cholestasis	1(2.3)	1(2.6)	1.00
Growth restriction	4 (9.1)	1 (2.6)	0.458
GDM	6 (13.6)	5 (13.2)	1.000
Hypertension	7 (15.9)	8 (21.1)	0.751
After term	24 (54.5)	22 (57.9)	0.936
Social	1 (2.3)	1 (2.6)	1.000
Isoimmunisation	1 (2.3)	0 (0)	—

^§^1 case excluded due to induction being cancelled after commencement.

**Table 2:** Maternal outcomes following Induction of labour with Prostin or Cervidil.

	Prostin (*N* = 44)	Cervidil (*N* = 38)	RR	95% CI	*P*
	Number (%)				
Failed induction	1 (2.3)	3 (7.9)	0.29	0.03–2.65	0.51
Amniotomy	27 (61.4)	24 (63.2)	0.97	0.69–1.36	1.00
Oxytocin infusion	29 (65.9)	24 (63.2)	1.04	0.76–1.44	0.98
Epidural analgesia	19 (43.2)	18 (44.4)	0.91	0.57–1.47	0.87
LSCS	12 (27.3)	16 (42.1)	0.65	0.35–1.19	0.24
Vaginal delivery	32 (72.7)	22 (57.9)	1.26	0.91–1.74	0.24
Instrumental Delivery^#^	14 (31.8)	4 (10.5)	3.0	1.1–8.4	**0.04**
NonInstrumental Delivery	18 (40.9)	18 (47.4)	0.86	0.53–1.41	0.72
LSCS or instrumental Delivery^#^	26 (59.9)	20 (68.4)	1.12	0.76–1.66	0.72
Mean time to deliver^§^(hrs)	21.1	29.6			**0.02**

LSCS: lower segment caesarean section.

^#^Vacuum extractor or obstetric forceps used to assist vaginal delivery.

^§^From application of induction agent until delivery of neonate.
